# T-reg transcriptomic signatures identify response to check-point inhibitors

**DOI:** 10.1038/s41598-024-60819-8

**Published:** 2024-05-06

**Authors:** María del Mar Noblejas-López, Elena García-Gil, Pedro Pérez-Segura, Atanasio Pandiella, Balázs Győrffy, Alberto Ocaña

**Affiliations:** 1 Translational Research Unit, Translational Oncology Laboratory, Albacete University Hospital, 02008 Albacete, Spain; 2 Unidad nanoDrug, Centro Regional de Investigaciones Biomédicas, Universidad de Castilla-La Mancha, 02008 Albacete, Spain; 3 Departamento Química Inorgánica, Orgánica y Bioquímica, Facultad de Farmacia de Albacete-Centro de Innovación en Química Avanzada (ORFEO-CINQA), Universidad de Castilla-La Mancha, 02008 Albacete, Spain; 4 Medical Oncology Department, Hospital Clínico Universitario San Carlos, Instituto de Investigación Sanitaria San Carlos (IdISSC), 28040 Madrid, Spain; 5 Instituto de Biología Molecular y Celular del Cáncer, CSIC, IBSAL and CIBERONC, 37007 Salamanca, Spain; 6 Department of Bioinformatics, Semmelweis University, Tűzoltó U. 7-9, Budapest, 1094 Hungary; 7 Research Centre for Natural Sciences, Hungarian Research Network, Magyar Tudosok Korutja 2, Budapest, 1117 Hungary; 8 Department of Biophysics, Medical School, University of Pecs, Pecs, 7624 Hungary; 9 Experimental Therapeutics Unit, Medical Oncology Department, Hospital Clínico Universitario San Carlos (HCSC), Instituto de Investigación Sanitaria (IdISSC) and CIBERONC and Fundación Jiménez Díaz, Unidad START Madrid, Calle Del Prof Martín Lagos, S/N, 28040 Madrid, Spain

**Keywords:** Regulatory T cells, Anti-PD1, Anti-CTLA4, Breast cancer, Transcriptomic analyses, Tumour immunology, Cancer, Computational biology and bioinformatics

## Abstract

Regulatory T cells (Tregs) is a subtype of CD4+ T cells that produce an inhibitory action against effector cells. In the present work we interrogated genomic datasets to explore the transcriptomic profile of breast tumors with high expression of Tregs. Only 0.5% of the total transcriptome correlated with the presence of Tregs and only four transcripts, *BIRC6, MAP3K2*, *USP4* and *SMG1*, were commonly shared among the different breast cancer subtypes. The combination of these genes predicted favorable outcome, and better prognosis in patients treated with checkpoint inhibitors. Twelve up-regulated genes coded for proteins expressed at the cell membrane that included functions related to neutrophil activation and regulation of macrophages. A positive association between *MSR1* and *CD80* with macrophages in basal-like tumors and *between OLR1, ABCA1, ITGAV, CLEC5A* and *CD80* and macrophages in HER2 positive tumors was observed. Expression of some of the identified genes correlated with favorable outcome and response to checkpoint inhibitors: *MSR1, CD80, OLR1, ABCA1, TMEM245*, and *ATP13A3* predicted outcome to anti PD(L)1 therapies, and *MSR1, CD80, OLR1, ANO6, ABCA1, TMEM245*, and *ATP13A3* to anti CTLA4 therapies, including a subgroup of melanoma treated patients. In this article we provide evidence of genes strongly associated with the presence of Tregs that modulates the response to check point inhibitors.

## Introduction

Cancer immune response depends on the interaction of the tumor with the host microenvironment^1,2^. This interaction dictates the immune reaction against the cancer and depends on different factors being one of them the genomic alterations that the tumor harbors^2,3^. Tumors with high genomic instability are those that produce more neoantigens and therefore prime antigen presenting cells that subsequently activate effector T cells^3^. However, oncogenic genomic alterations like HER2 amplification, or mutations at the EGFR or BRAF gene, among others, induce phenotypical changes that modulates the immune response, leading in some occasions, to an inhibited immune microenvironment^4^.

Several cells participate in the host immune response against cancer. For instance, the presence of tumor infiltrating lymphocytes (TILs) is associated with better outcome in some cancer types^5,6^. Similarly, the reduction of inhibitory signals acting with antibodies against PD1 or its ligand PDL1 has shown to enhance the immunologic effect against cancer, effect that has translated into clinical benefit^7,8^.

A subset of cells with inhibitory functions is the one termed regulatory T cells (Tregs). Tregs belong to the CD4+ T cell subtype that also includes Th1, Th2, Th17 and follicular helper T cells^9,10^. Tregs account for 5–10% of total peripheral CD4+ T cells and are characterized by the presence of the transcription factor FOXP3^11^. Tregs play a central role inhibiting the immune response against tumors by secreting several immunosuppressive factors^12^. These cytokines inhibit effector T and NK cells; and promote tumoral M2 macrophages^13^. Several strategies have been pursued to inhibit their activity by acting on cell surface molecules that regulate their function including CD25, CTLA4, CD36, among others^14^. In this context, targeting some of these proteins like CTLA4 with antibodies has shown benefit in patients^15,16^. In addition, clinical studies targeting some of these molecules are under evaluation in early phase trials including compounds against GITR, CD25 or OX40, among others^17^.

Efficient antitumor immune activation requires the effect against different targets to enhance a multicell effector action. This has been demonstrated with the combination of anti PD(L)1 inhibitors with CTLA4 inhibitors in several indications like melanoma or MSIH colorectal cancer^15,16^. In this context, identification of targets that are expressed simultaneously is mandatory to design smart drug combinations. In a similar way, the discovery of markers of response will undoubtedly permit the administration of therapies to resistant patients. In this context, proteins expressed at the surface of the membrane are attractive targets or markers, as are easily accessible with antibodies against them: therefore, mapping the cell surfaceome is a therapeutic priority in drug development.

In our study we aimed to evaluate the immune transcriptomic profile of tumors that harbor high presence of Tregs. Our goal was to identify genomic vulnerabilities linked to the presence of Tregs that could be druggable pharmacologically. In addition, we explored transcriptomic signatures of response to agents targeting Tregs like CTLA4 antibodies.

## Results

### Mapping upregulated genes in breast cancer tumors expressing Tregs

**Figure 1:**
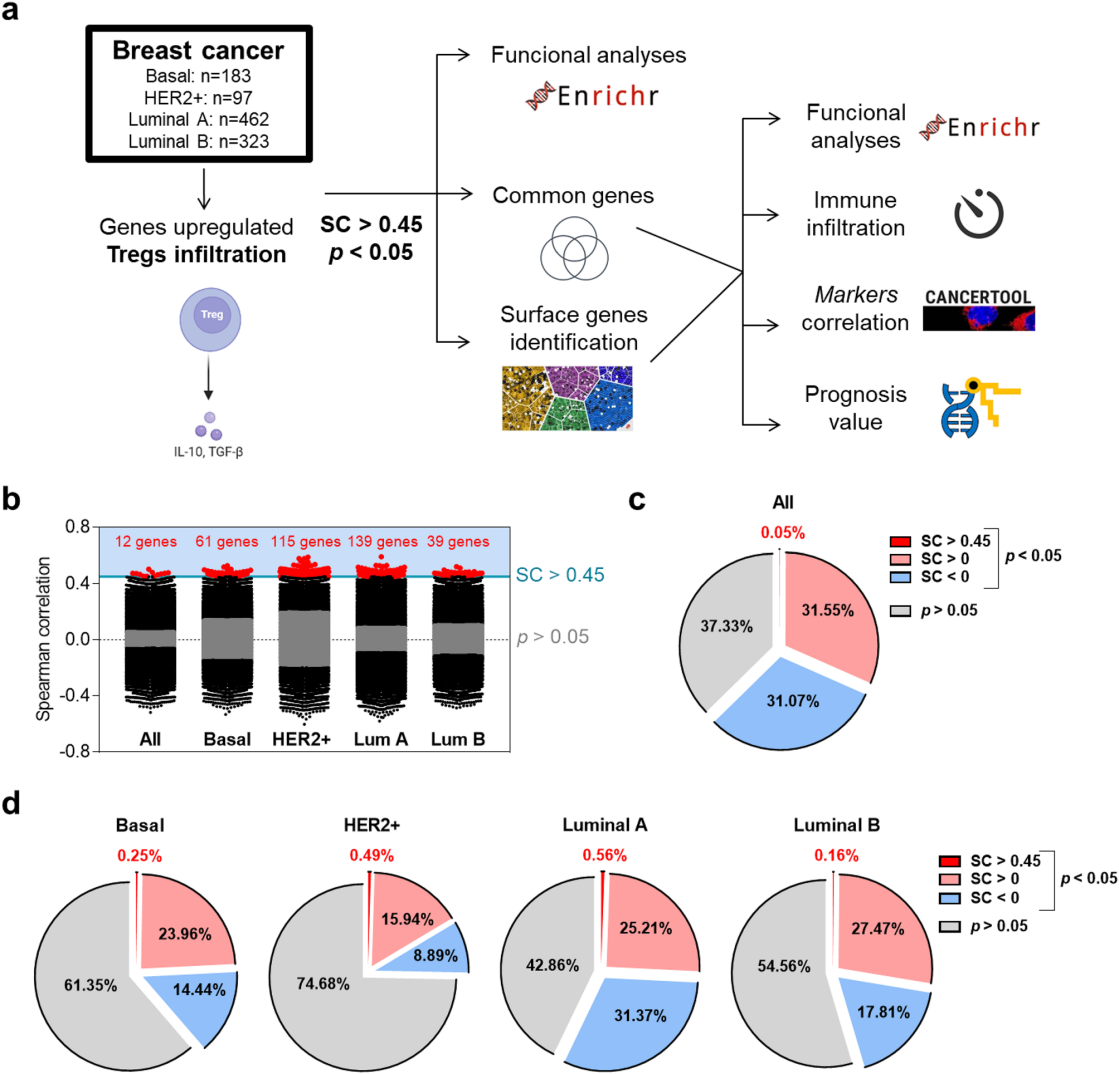
Identification of up-regulated genes associated with Tregs infiltration. (**a**) Flow chart describing the results obtained during the process and the bioinformatic analysis used. (**b**) Genes with spearman correlation > 0.45 were considered as positively correlated with Treg infiltration. Pie chart displaying the proportion of genes with different SC in whole breast cancer group (**c**) and by subtypes (**d**).

To identify upregulated genes expressed in tumors with high presence of regulatory T cells (Tregs) we interrogated public datasets, as described in the material and method section. Figure 1a displays the flow chart of the whole analysis. Using a correlation score (threshold of spearman rank correlation) > 0.45 with a p < 0.05 (for statistical analysis see material and methods section) (Fig. 1a), we identified twelve genes correlated in the entire breast cancer population with high Treg expression. When performing the analysis in breast cancer subtypes independently, we recognized sixty-one genes in basal-like tumors, one hundred fifteen genes in HER2 positive tumors, and one hundred thirty-nine and thirty-nine genes in the Luminal A and B subtype, respectively (Fig. 1b, Supplementary Table 2). Figure 1c,d describe the proportion of the selected genes within all transcriptome: 0.5% of genes in the entire population, 0.25% in the basal-like, 0.49% in HER2, and 0.56% and 0.16% in Luminal A and Luminal B molecular subtypes, respectively. These data suggest that the described genes constituted a minority of the entire transcriptomic profile. Functional analysis of the identified genes is fully represented in Supplementary Fig. 1.

### Common up-regulated genes among different subtypes and association with immune populations

**Figure 2:**
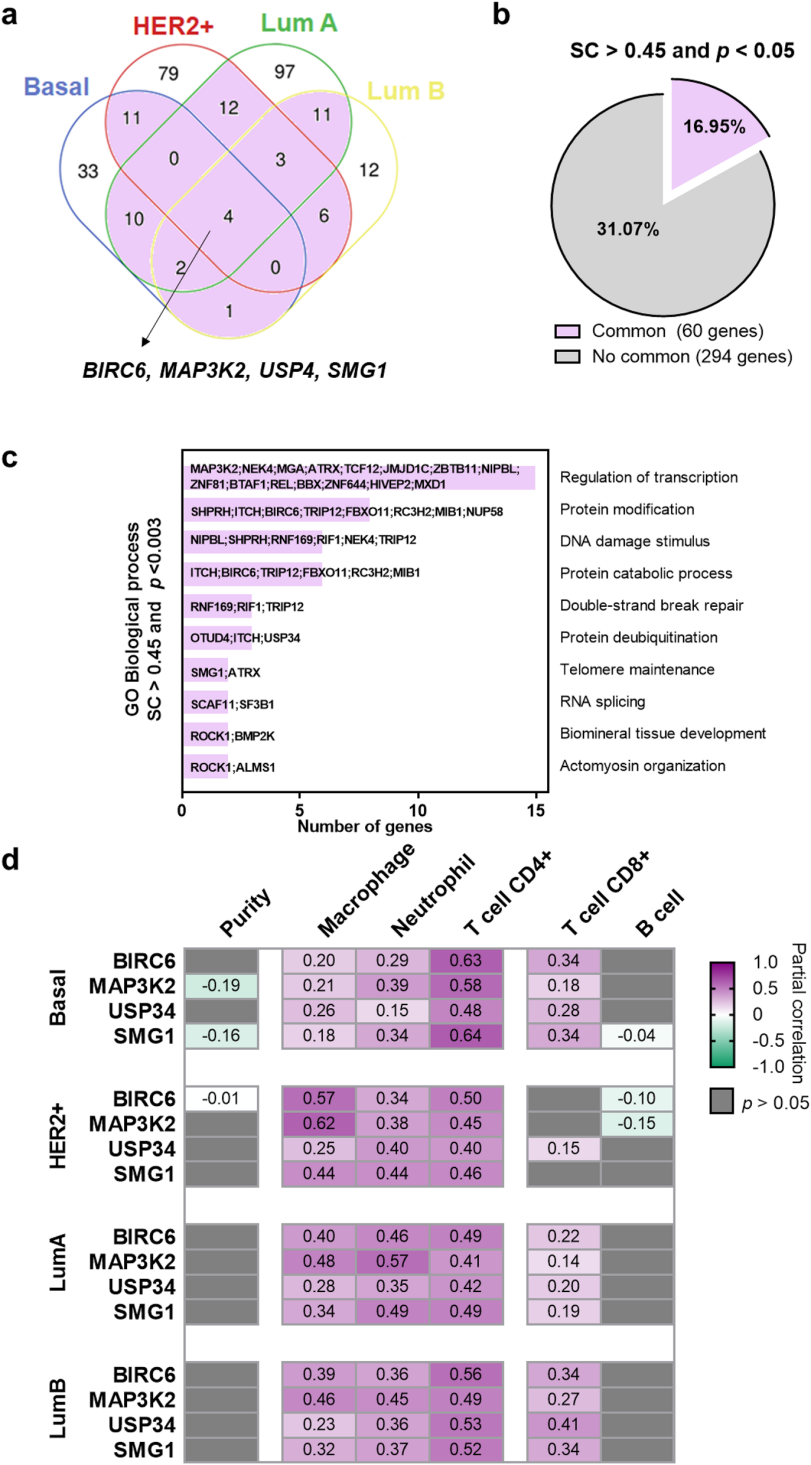
Evaluation of common genes between subtypes. (**a**) Venn diagram including genes with SC > 0.45 in breast cancer subtypes. (**b**) Pie chart with proportion of common genes in at least two subtypes. (**c**) Functional analyses by Enrichr of sixty common genes (included in two subtypes or more). (**d**) Heat map depicting the Pearson correlation coefficient (R) between gene expression, tumor purity, and the presence of tumor immune infiltrates in breast cancer subtypes.

Only four genes were commonly present in all breast cancer subtypes, and those included *BIRC6, MAP3K2, USP4* and *SMG1*, as can be seen in Fig. 2a. Sixty (16.95%) genes were shared among any of the subtypes (Fig. 2b) and 51 (85.00%) were common in two subtypes (Supplementary Fig. 2A). The HER2+ and the Luminal A subtype, were the combo-subtype that shared more genes between both groups (23.53%) (Supplementary Fig. 2B), followed by the Luminal A and Luminal B, and finally the Basal-HER2+ combo-group (21.57%). We evaluated if these genes *BIRC6, MAP3K2, USP4* and *SMG1*, coded for proteins, so we explored their presence using The Human protein Atlas (Supplementary Fig. 3), confirming their presence.

Functional analysis of commonly shared up-regulated genes revealed Regulation of transcription, Protein modification and DNA damage stimulus, among the most present functions, as can be seen in Fig. 2c.

We next correlated the expression of commonly shared identified genes; *BIRC6, MAP3K2, USP4* and *SMG1* with immune populations in different breast cancer subtypes. Doing so we aimed to identify additional immune populations present within the immune microenvironment. As expected, a positive correlation was observed between the expression of these genes and CD4+ T cell populations in all subtypes (Fig. 2d). A lower correlation was identified for macrophages, neutrophils and CD8+ T cells and no presence of B cells were observed. This set of data could suggests that these genes are present in tumors with high expression of CD4+ T cells, but also in other populations that could be susceptible for immune modulation. Interestingly, BIRC6 and MAP3K2 highly correlated with macrophages in the HER2+ subtype (Fig. 2d). A complete evaluation of the different T cell populations is displayed in Supplementary Figs. 4 and Supplementary Fig. 5. Of note the CD4+ T cells consisted mainly of memory T cells and were the population more associated with the expression of these genes.

### Surfaceome proteins correlated with macrophages and PD-L1 expression

**Figure 3:**
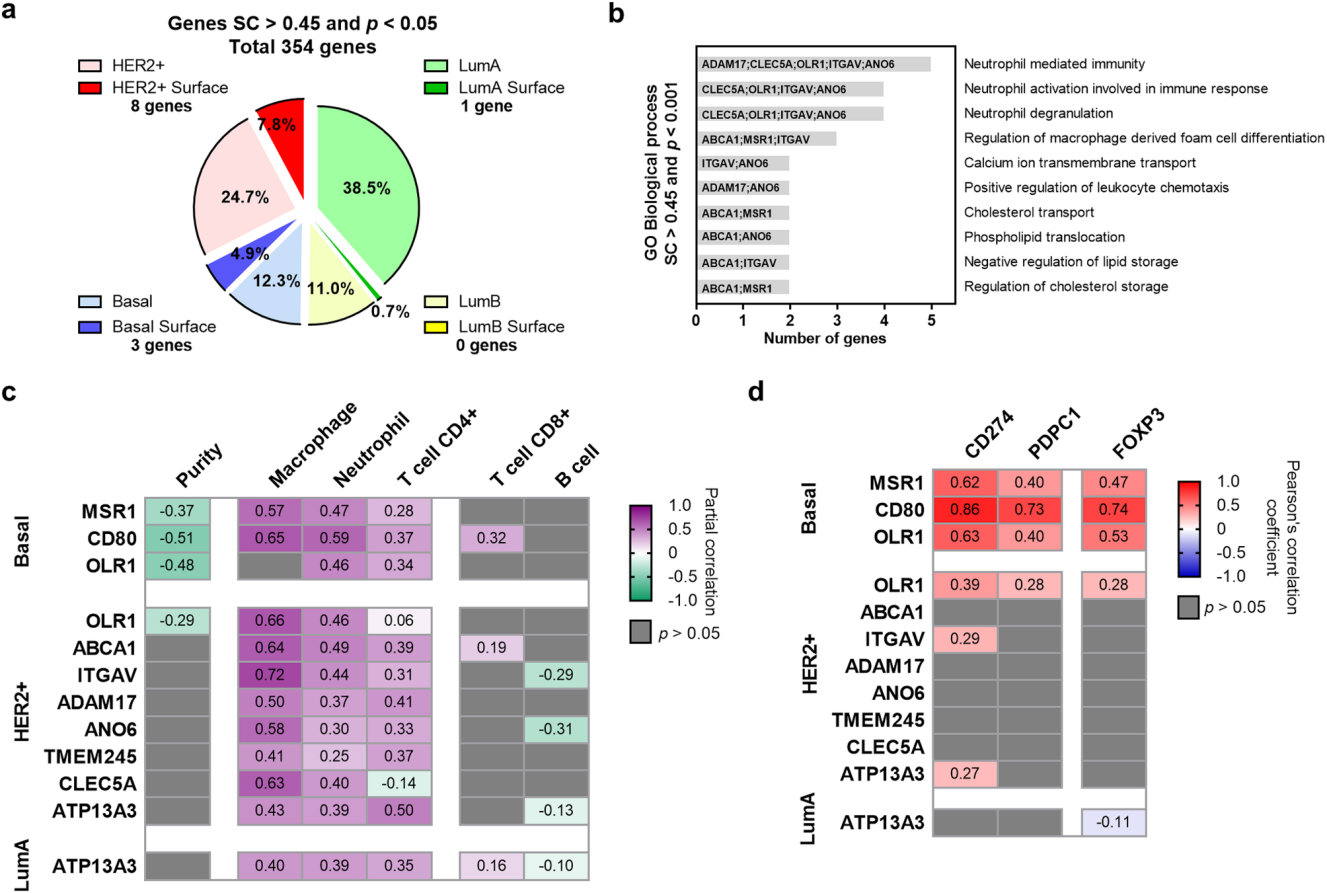
Evaluation of surfaceome genes. (**a**) Pie chart with proportion of surfaceome genes by subtypes. (**b**) Functional analyses of the surfaceome genes performed by Enrichr. (**c**) Heat map depicting the Pearson correlation coefficient (R) between gene expression, tumor purity, and the presence of tumor immune infiltrates in breast cancer subtypes. (**d**) Heat map depicting the Pearson correlation coefficient (R) of the association between macrophage markers and the expression of the selected genes using CANCERTOOL and the TCGA cohort.

Ten up-regulated genes coded for proteins expressed at the cell membrane, being eight of those (7.8%) from the HER2 subtype, one (0.7%) in the Luminal A, and three (4.9%) genes from the basal-like subtype (Fig. 3a). No genes were identified in Luminal B subtype. Functional analyses of these genes displayed ontologies related to the immune system as Neutrophil mediated immunity, Neutrophil activation, Neutrophil degradation; or Regulation of macrophage (Fig. 3b).

We observed a positive correlation between *MSR1* and *CD80* with macrophages in basal-like tumors and between *OLR1*, *ABCA1*, *ITGAV*, *CLEC5A* and *CD80* and macrophages in HER2 positive tumors (Fig. 3c). A very strong correlation was observed between *CD80* and *CD274*/PDL1 and *PDPC1*/PD1, and a strong association with FOXP3 indicative of the presence of *CD80* mainly in Tregs (Fig. 3d). The association was weaker for *OLR1* and *MSR1*. In other breast cancer subtypes, no clear association was identified when evaluating all markers with *CD274*/PDL1, *PDPC1*/PD1 and FOXP3 (Fig. 3d). These results suggest that proteins coded by the described surfaceome genes are expressed in cells within the tumor microenvironment including, but not limited to, macrophages particularly in basal-like tumors. Supplementary Table 3 provides a full list of the described genes and an explanation of their biological role.

### Association of the identified genes with clinical outcome

In a next step we explored the presence of the reported genes with patient clinical outcome including relapse free survival (RFS) and overall survival (OS).

**Figure 4:**
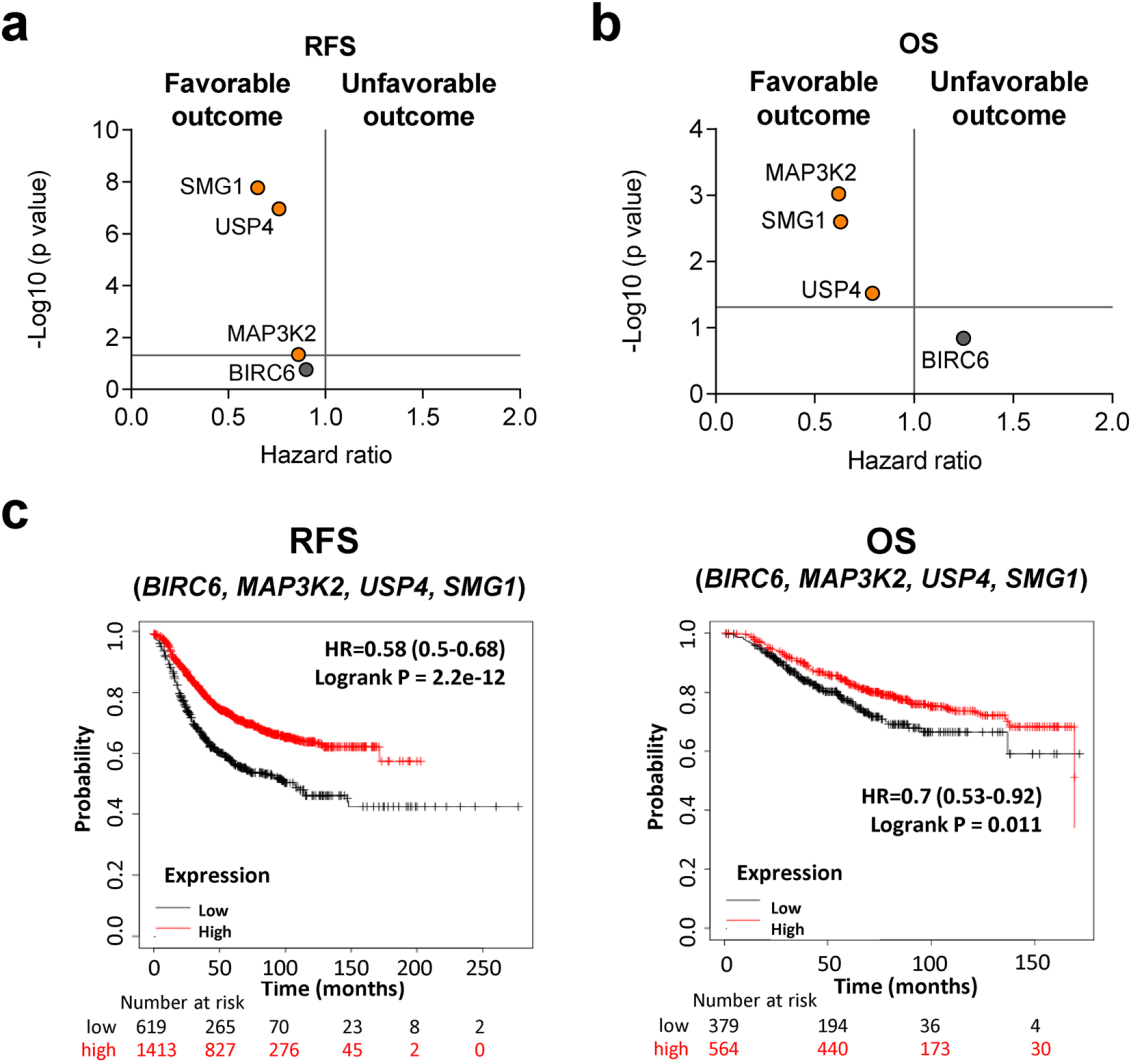
Common up-regulated genes associated with outcome in breast cancer. Dot plot displaying HR values extracted from Kaplan–Meier survival plots of the association between common genes individually expressed and patient prognosis, including relapse-free survival (RFS) (**a**) and overall survival (OS) (**b**), for all breast cancer patients from the exploratory cohort. (**c**) Kaplan–Meier survival plots of the association between common genes mean expression levels and patient prognosis, for all subtypes including RFS and OS. All: n = 2032 (RFS) and n = 253 (OS).

For common genes, individually *MAP3K2, SMG1* and *USP4* displayed a favourable outcome in both RFS and OS analyses, however *BIRC6* showed no significative association with outcome (Fig. 4a,b). As a signature, these genes showed a statistically significant association with favourable survival (RFS: HR = 0.58; CI 0.5–0.68; p = 2.2 × 10–12; OS: HR = 0.70; CI 0.53–0.92; p = 0.011) (Fig. 4c).

**Figure 5:**
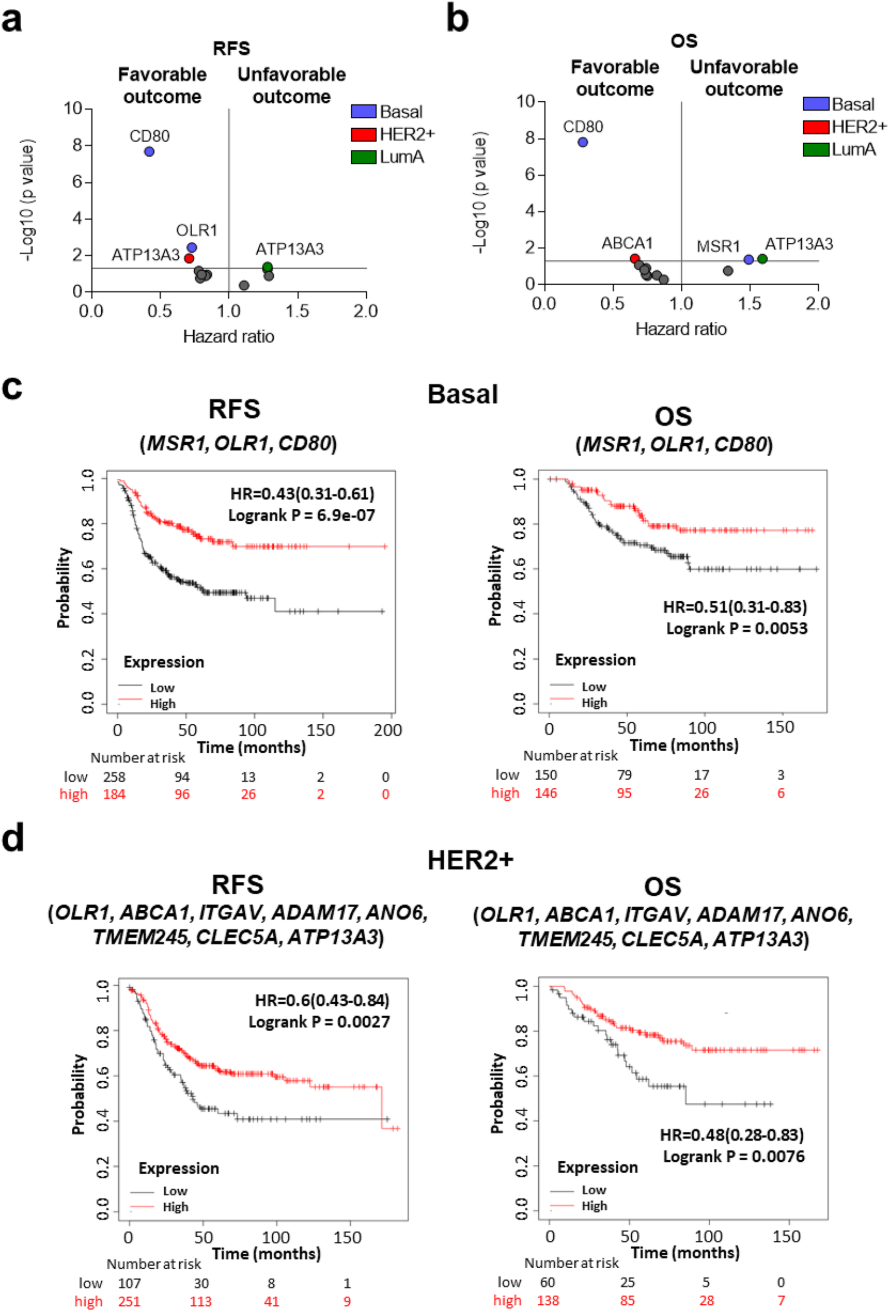
Surface related transcriptional profiles associated with outcome in breast cancer. Dot plot displaying HR values extracted from Kaplan–Meier survival plots of the association between surface genes individually expressed and patient prognosis, including relapse-free survival (RFS) (**a**) and overall survival (OS) (**b**), for all breast subtypes from the exploratory cohort. Kaplan–Meier survival plots of the association between surface genes mean expression levels and patient prognosis, for basal (**c**) and HER2 + (**d**) subtypes including RFS and OS. Basal-like: n = 442 (RFS) and n = 296 (OS); HER2 + : n = 358 (RFS) and n = 198 (OS) and Luminal A: n = 1809 (RFS) and n = 596 (OS).

For those genes expressed at the surfaceome of cells, individually, only *CD80* expression in basal subtype had a profound favourable outcome (RFS: HR = 0.42; CI 0.31–0.58; p = 2.1 × 10–8; OS: HR = 0.28; CI 0.18–0.45; p = 1.5 × 10–8) (Fig. 5a,b). Of note, when we used these genes as a signature using a mean of expression of all genes divided by subtype, we observed a high association with favourable outcome in the basal-like (RFS: HR = 0.43; CI 0.31–0.61; p = 6.9 × 10–7; OS: HR = 0.51; CI 0.31–0.83; p = 0.0053) (Fig. 5c) and HER2+ (RFS: HR = 0.6; CI 0.43–0.84; p = 0.0027; OS: HR = 0.48; CI 0.28–0.83; p = 0.0076) breast cancer subtypes (Fig. 5d).

### Presence of genes and response to anti PD (L)1 and CTLA4 antibodies

Finally, we intended to study the presence of the described genes with response to PD1 or CTLA4 therapies. To do so we collected data as described in the material and methods section.

**Figure 6:**
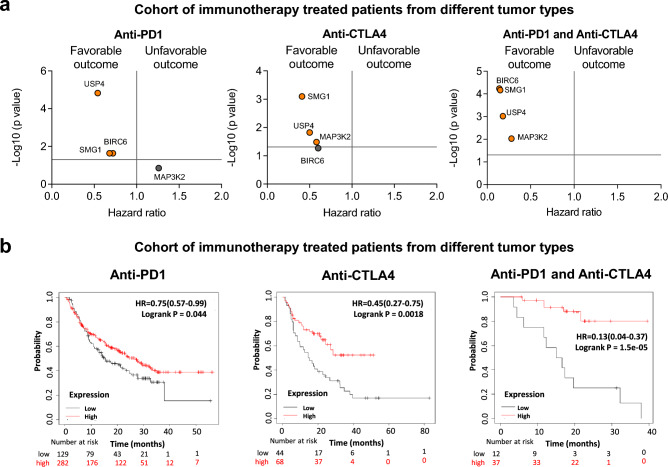
Common up-regulated genes associated with outcome in patients treated with anti-PD1 and anti-CTLA4. (**a**) Dot plot displaying HR values extracted from Kaplan–Meier survival plots of the association between common genes individually expressed and patient prognosis conditionate to anti-PD1 (left), anti-CTLA4 (middle) and both (right) treatments, for all exploratory cohort. (**b**) Kaplan–Meier survival plots of the association between common genes mean expression levels and patient prognosis conditionate to anti-PD1, anti-CTLA4, and both treatments. Anti-PD1 n = 797 and Anti-CTLA4 n = 131. Cohort of immunotherapy treated patients from different tumor types including bladder (n = 90), esophageal adenocarcinoma (n = 103), glioblastoma (n = 28), hepatocellular carcinoma (n = 22), HNSCC (n = 110), melanoma (n = 570), NSCLC (n = 21), NSLC (n = 22), breast (n = 14), gastric (n = 45) and urothelial (n = 392).

Favorable outcome was observed in patients treated with anti-PD1 for *BIRC6, USP4 and SMG1* (Fig. 6a, left panel). For anti CTLA4 therapies, an association with better survival was observed for the genes *MAP3K2, USP4 and SMG1* (Fig. 6a, middle panel). Lastly, when we evaluated the effect on survival in patients treated with both agents, we observed a positive association with the four common genes (Fig. 6a, right panel).

When we used these genes as a signature using a mean of expression of the four genes, we observed an association with favorable outcome, for anti-PD1, anti-CTLA4 or combinatorial therapies, highlighting its predictive role for both treatments (Anti-PD1 + Anti-CTLA4: HR = 0.13; CI 0.04–0.37; p = 1.5 × 10–5) (Fig. 6b).

**Figure 7:**
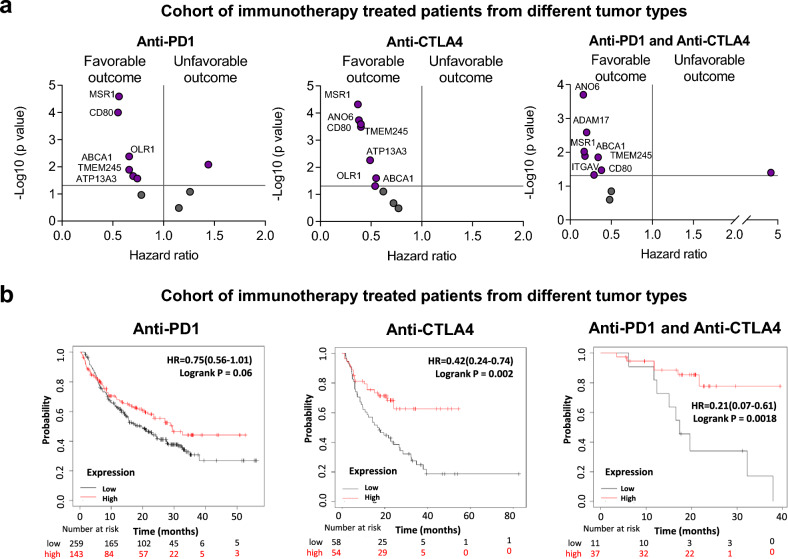
Surface genes associated with outcome in patients treated with anti-PD1 and anti-CTLA4. (**a**) Dot plot displaying HR values extracted from Kaplan–Meier survival plots of the association between surface genes individually expressed and patient prognosis conditionate to anti-PD1 (left), anti-CTLA4 (middle) and both (right) treatments, for all exploratory cohort. (**b**) Kaplan–Meier survival plots of the association between surface genes mean expression levels and patient prognosis conditionate to anti-PD1, anti-CTLA4, and both treatments. Anti-PD1 n = 797 and Anti-CTLA4 n = 131. Cohort of immunotherapy treated patients from different tumor types including bladder (n = 90), esophageal adenocarcinoma (n = 103), glioblastoma (n = 28), hepatocellular carcinoma (n = 22), HNSCC (n = 110), melanoma (n = 570), NSCLC (n = 21), NSLC (n = 22), breast (n = 14), gastric (n = 45) and urothelial (n = 392).

For surfaceome genes, for anti PD1 therapies a trend towards a better outcome was observed for *MSR1*, *CD80*, *OLR1*, *ABCA1*, *TMEM245*, and *ATP13A3* (Fig. 7a, left panel). Similarly, for anti CTLA4 therapies, an association with better survival was observed for the genes *MSR1*, *CD80*, *OLR1*, ANO6, *ABCA1*, *TMEM245*, and *ATP13A3* (Fig. 7a, middle panel). We also evaluated the effect on survival in patients treated at the same time with both agents. In this case better outcome was observed for the genes *ANO6, ADAM17*, *MSR1*, *CD80*, *ITGAV*, *ABCA1*, and *TMEM245* (Fig. 7a, right panel).

When we used these genes as a signature using a mean of expression of all genes (10 genes), we observed an association with favourable outcome, being statistically borderline significant for PD1 treatment, and the association was strongly positive for anti-CTLA4 or combinatorial therapies (Anti PD1: HR = 0.75; CI 0.56–1.01; p = 0.06; Anti CTLA4: HR = 0.42; CI 0.24–0.74; p = 0.002; Anti PD1 + Anti CTLA4: HR = 0.21; CI 0.07–0.61; p = 0.0018) (Fig. 7b). Also, this association was observed when we restrict the analysis to pre-treated or on-treated patients. Except anti-PD1 in pre-treatment samples (Supplementary Fig. 6).

Of note, we also studied the association of described genes with response to Ipilimumab (anti-CTLA4 therapy), in melanoma patients. As shown in Supplementary Fig. 7, better survival was observed for the genes *MSR1*, *CD80*, *OLR1*, *ABCA1*, ANO6, *TMEM245*, and *ATP13A3*.

The capacity of the genes to predict response to immunotherapy was further confirmed using a different cohort of patients treated with anti-PD1 or anti-CTLA4. This dataset is not limited to breast cancer and includes different solid tumor types as described in Material and Methods section.

**Figure 8:**
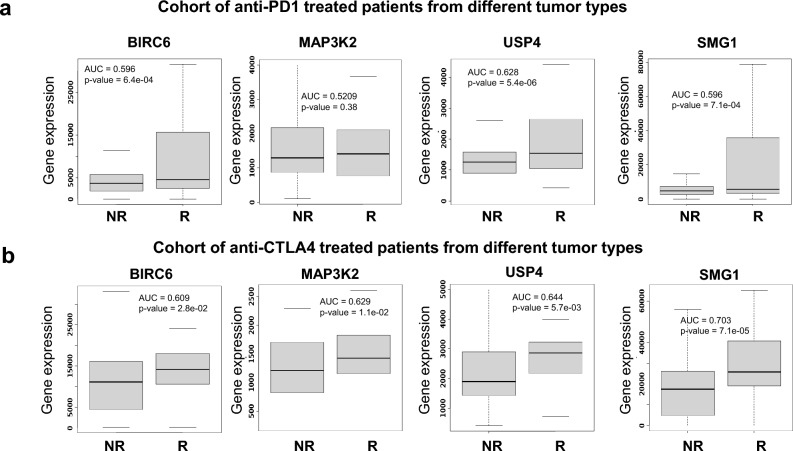
Common up-regulated genes associated with response in patients treated with anti-PD1 and anti-CTLA4. Box-plots of genes validated for Anti-PD1 response in (**a**) or Anti-CTLA4 response (**b**) in cancer patients using the pathological complete response database in ROC plotter. Graphs show normalized gene expression in non-responders (NR) and responders (R) patients. Cohort of different solid tumors that include metastatic and primary tumors treated with immunotherapy.

In patients who responded to anti-PD1 therapies the expression of these genes in pretreated samples predicted response: *BIRC6* (AUC = 0.596, p = 6.4e − 04), *USP4* (AUC = 0.628, p = 5.4e − 06), and *SMG1* (AUC = 0.596, p = 7.1e − 04) (Fig. 8a). Similarly, the expression of the four genes was higher in patients who responded to anti-CTL4A therapies (Fig. 8b).

**Figure 9:**
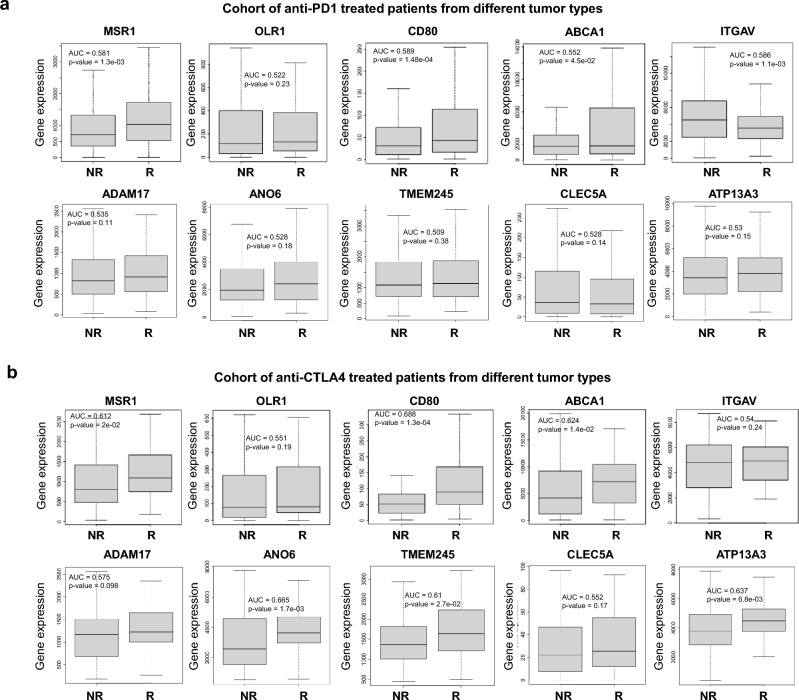
Surface genes associated with response in patients treated with anti-PD1 and anti-CTLA4. Box-plots of genes validated for Anti-PD1 response in (**a**) or Anti-CTLA4 response (**b**) in cancer patients using the pathological complete response database in ROC plotter. Graphs show normalized gene expression in non-responders (NR) and responders (R) patients. Cohort of different solid tumors that include metastatic and primary tumors treated with immunotherapy.

The expression of *MSR1* (AUC = 0.581, p = 1.3e − 03), *CD80* (AUC = 0.589, p = 1.48e − 04), and *ABCA1* (AUC = 0.552, p = 4.5e − 02), was higher in patients who responded to anti-PD1 therapies (Fig. 9a). Meanwhile, the expression of *MSR1* (AUC = 0.612, p = 2e − 02), *CD80* (AUC = 0.688, p = 1.3e − 04), *ABCA1* (AUC = 0.624, p = 1.4e − 02), ANO6 (AUC = 0.665, p = 1.7e-03), *TMEM245* (AUC = 0.61, p = 2.7e − 02) and *ATP13A3* (AUC = 0.637, p = 6.8e − 03) was also higher in patients who responded to anti-CTLA4 therapies (Fig. 9b). These results were obtained exclusively from pre-treatment samples. When we compared the evaluation with on-treatment samples we obtained the same trend (Supplementary Fig. 8).

## Discussion

In the present article we explore the transcriptomic profile of tumors that harbor high expression of Tregs with the final aim to identify genomic correlates of response to check point inhibitors and potential druggable vulnerabilities.

Tregs are a subpopulation of CD4+ T cells that constitute around 4–9% of this cell population^18^. Their principal role is related to the inhibition of the effector immune cell response mediated by activated CD8+ T cells favoring T cell exhaustion^9,19^. In cancer, several studies have demonstrated that this population plays a central role mediating tumor progression, and indeed inhibition of their effect acting on the CTLA4 receptor has shown to increase survival in several tumor types^20^. In addition, other therapies aiming to act on receptors expressed in this population are in late stage of clinical development like those targeting TIGIT^21^.

When evaluating upregulated genes that associated with high presence of Tregs we observed that only a minority of genes strongly correlated with this population being only 0.5% of all genes in the whole population. In our analysis we used a double approach, we first explored highly upregulated genes and secondly, we focused only on those present at the membrane of the cells. Only sixty genes (17%) were commonly shared between breast cancer subtypes, being only four of them present in the four subtypes: *BIRC6, MAP3K2, USP4* and *SMG1*. Functions of those genes included Regulation of transcription, Protein modification and DNA damage stimulus. The common four genes were associated with the presence of CD4+ T cells and *CD274*/PDL1, suggesting that their presence was not restricted to a population of Tregs^11^. The identified signature predicted favorable outcome in breast cancer patients and better prognosis in patients treated with checkpoint inhibitors.

In a next step, we focused only of those genes located at the plasma membrane. Twelve genes were identified and functionally linked with neutrophil mediated immunity and macrophage regulation. *MSR1* and *CD80* correlated with macrophages in basal-like tumors and between *OLR1*, *ABCA1*, *ITGAV*, *CLEC5A* and *CD80* and macrophages in HER2 positive tumors.*CD80* associated with *CD274*/PDL1 and FOXP3 in basal-like tumors suggesting that this biomarker can be expressed in different immune populations that co-exist within the same tumor microenvironment. *CD80* has been expressed in different immune cells in a tumor type context dependent, mainly in cells with antigen presenting functions^22,23^. In addition, expression of *CD80* has been considered as necessary to sustain Treg populations^24^. In this context some studies have found that mice lacking *CD80* had a decreased number of Tregs in the thymus and periphery predisposing to autoimmune disease^23^. Finally, in line with this, CD28, the receptor of *CD80*, is necessary for the production of Tregs. Although *CD80* has been associated with Treg modulation as described before^24^, in our analysis we observed an association with favorable outcome in untreated patients. Although this is somehow a contradictory finding, an association with favorable prognosis has also been observed for high expression of inhibitory receptors and ligands like PD1 or PDL1^25,26^. This suggests that an immune reactive but suppressed microenvironment is present. The association with *OLR1* and *MSR1* was weaker. *CD80* or CTLA4 is a known co-inhibitory receptor present in Tregs and antibodies targeting this protein like ipilimumab has shown to produce clinical activity. *MSR1* has been described as present in M2 macrophages contributing to inflammation and patient outcome^27,28^. Indeed, presence of *MSR1* has been linked with T cell exhaustion and it has been included in a gene signature that predicted favorable response to anti PD (L)1 in liver cancer^29^. *MSR1* is a gene that codes for a membrane glycoprotein implicated in the pathologic deposition of cholesterol in arterial walls during atherogenesis and mediates the endocytosis of a diverse group of macromolecules, including modified low-density lipoproteins (LDL)^30^. A more detailed description of the biological role of the identified surfaceome genes is provided in Supplementary Table 3.

Finally, we observed that some of the identified genes correlated with favorable prognosis and response to anti PD1 and CTLA4 therapies. The selected gene signature defined outcome in basal-like and HER2 tumors for RFS and OS. When evaluating patients treated with check point inhibitors, the selected gene signature correlated with clinical response and a favorable survival, and this was clearly observed for anti CTLA4 and both anti PD1 and anti CTLA4 agents. When focus on Ipilimumab, particularly in melanoma, similar findings were observed. Finally, some genes specifically correlated with response to Ipilimumab like *MSR1*, *CD80*, *OLR1*, *ABCA1*, *ANO6*, *TMEM245*, and *ATP13A3*. The presence of *MSR1* suggest the relevant role of macrophages modulating the inhibitory effect of Tregs. Finally, we confirmed how the presence of some genes predicted response to anti PD1 and or anti CTLA4 in combination with chemotherapy in the neoadjuvant setting. This data highlights that for a selected number of genes a short treatment course, as that given in the neoadjuvant setting, is enough to predict response to these immunotherapy agents.

In our article we identify a set of different genes that are probably expressed in a wide range of cells, mainly CD4+ T cell, macrophages, and neutrophils. Of note, none of these genes is characteristic of a specific immune population, and therefore could be expressed in a range of immune cells. Their presence was more clearly identified in the basal-like subtype where an association with PDL1 was also observed. These findings suggest that there is an immune-repressed microenvironment that clearly favors the activity to CPIs.

Several articles have described immune signatures in breast cancer, but only few describe the association between the presence of Tregs and outcome^31–35^. However, no evaluation of the transcriptomic profile in relation to the presence of immune cell populations, including Tregs, has been performed in this indication. Although several gene signatures have been described in relation to response to check point inhibitors mainly anti PD(L)1 agents^36^, little has been reported about the activity of both anti-PD(L)1 agents and the anti CTLA4 antibody ipilimumab. We also acknowledge that this is a bioinformatic analysis and the use of other techniques like spatial transcriptomics, single cell analysis or direct evaluation of protein expression with immunohistochemistry techniques will undoubtedly had enriched the manuscript^37,38^.

In summary, we describe transcriptomic correlates present in breast tumors with high expression of Tregs, identifying a gene signature that predicts clinical benefit of the approved check-point inhibitors PD (L)1 and CTLA4 antibodies. The described signature in the manuscript is protected by the following patent application: EP23382324. The relevant role of Tregs in suppression of T lymphocyte action on tumoral cells opens the possibility of acting on the former to restore T cell fitness against tumors. Identification of manners of controlling Treg action may therefore augment immune anti-tumoral responses. In this respect, the data presented here uncovers potential options to optimize these treatments.

## Methods

### Identification of genes related with Tregs infiltration and functional analyses

Breast cancer samples including patients from datasets described previously^39–41^ were used as a cohort to identify genes whose expression correlated to high regulatory T-cells (Tregs) infiltration. Immune cell infiltration for each tumor sample was determined by using the normalized RNA-seq based transcriptome-wide gene expression data as input for the xCell algorithm^42^. xCell is designed to compute surrogate markers of cellular proportions for all together sixty-four different cell types. Then, Spearman rank correlation was computed for each gene to compare its normalized gene expression and the xCell derived infiltration scores for regulatory T-cells. High value as a Treg score corresponds to higher proportion of Treg cells among all cells in the entire bulk tumor sample. Finally, all investigated genes were ranked based on the achieved Spearman correlation coefficients. The analysis was performed in all patients and in each of the molecular subtypes independently—the molecular subtypes were determined using the PAM50 signature and include basal (lacking ER, PGR, and HER2 expression) n = 183), Luminal A (ER or PR positive with low KI67 expression, n = 462), Luminal B (ER or PR positive with high KI67 expression, n = 323), and HER2 enriched (HER2 positive, n = 97) cohorts. The correlation analysis included a total of 25,229 genes. To elucidate common upregulated genes associated with Treg infiltration in some breast cancer subtypes Venn diagrams were performed. We followed procedures described at: http://bioinformatics.psb.ugent.be/webtools/Venn/.

Genes that correlated with Tregs infiltration were analyzed using the biological function enrichment analyses tool Enrichr^43^. We compilated Biological process or Molecular function ontologies (2021 version) with a determinate p-value indicative of each functional study (< 0.05 in all cases).

### Surface protein identification

We applied the in silico human surfaceome^44^ to identified genes that encode surface proteins. This public biomedical resource can be used to filter multiomics data to uncover cellular phenotypes and new surfaceome markers.

### Association with tumor immune infiltrates

Tumor Immune Estimation Resource (TIMER) platform^45^ was employed to analyze tumor purity, and the association between the presence of tumor immune infiltrates (CD4+ T cells, CD8+ T cells, macrophages, neutrophils, and B cell). TIMER contains 10,897 samples from diverse cancer types from the TCGA (The Cancer Genome Atlas) project and provides immune infiltrates' abundances estimated by multiple immune deconvolution methods. TIMER applies a deconvolution method previously published^46^ to infer the abundance of tumor-infiltrating immune cells from gene expression profiles. For estimation of cell type abundances from bulk tissue transcriptomes by CIBERSOFT multiple hypothesis testing was performed using the Benjamini and Hochberg method^47^. We explored the tumor immune infiltrates in breast cancer subtypes.

### Outcome analyses and gene correlations

KM Plotter Online Tool^39–41^ was used to evaluate the relationship between the expression of the genes and patient clinical prognosis. This database permits the evaluation of relapse-free survival (RFS) and overall survival (OS) in breast tumors by subtypes. For outcome analyses, patients were separated according to auto best cut-off values. Patients above the threshold were deemed “high” expression while patients below the threshold were characterized as “low” expression. The number of samples included in HGU133 array 2.0 for each subtype was: all: n = 2032 (RFS) and n = 953 (OS); basal-like: n = 442 (RFS) and n = 296 (OS); HER2 + : n = 358 (RFS) and n = 198 (OS) and Luminal A: n = 1809 (RFS) and n = 596 (OS).

In an independent Kaplan–Meier analysis we correlated the gene expression and survival in a combined cohort of immunotherapy treated patients from different tumor types including bladder (n = 90), esophageal adenocarcinoma (n = 103), glioblastoma (n = 28), hepatocellular carcinoma (n = 22), HNSCC (n = 110), melanoma (n = 570), NSCLC (n = 21), NSLC (n = 22), breast (n = 14), gastric (n = 45) and urothelial (n = 392). The datasets were identified in GEO using the keywords “gene expression”, “PD1”, “CTLA4”, and “immunotherapy” as well as the names of available immunotherapy agents. In this cohort we evaluated the correlation to overall survival (OS) only and patients were also separated into two cohorts according to the best cut off values. According to administered therapy, anti-PD1 treatment included n = 797 samples and the anti-CTLA4 cohort included n = 131 samples.

The Kaplan–Meier (KM) plots are presented with the hazard ratio (HR), the 95% confidence interval (CI) and the log-rank p-value (p). Genes or signatures with a HR < 1, p < 0.05 were considered predictors of favorable outcome, while genes with a HR > 1, p < 0.05 were considered predictors of detrimental outcome.

The ROC plotter online tool^48^ was used to correlate gene expression and response to immunotherapy (anti-PD1 or anti-CTLA4) in an independent cohort of different solid tumors that include metastatic and primary tumors. The area under the curve (AUC) was computed to evaluate the clinical activity of the biomarker candidates. AUC values are independent of the used cut-off. This dataset, a public available tool, has been developed by some of the authors of this publication.

For correlation analysis between genes, we used the Pearson correlation coefficients of every pair of genes. Data from TCGA (The Cancer Genome Atlas)^49^ were included in the analysis.

Complete information describing all datasets used in the work is provided in the Supplementary Table 1.

### Supplementary Information

Supplementary Information.

## Data Availability

The datasets presented in this study can be found in online repositories. The names of the repository/repositories and accession number(s) can be found in the article/supplementary material. The data that support the findings of this study are available from the corresponding author upon reasonable request.
