# Supplementary material for: T-reg transcriptomic signatures identify response to check-point inhibitors
Source: Sci Rep. 2024 May 6;14:10396. doi: 10.1038/s41598-024-60819-8 (PMC11074113; doi:10.1038/s41598-024-60819-8)
Supplement: Supplementary file 1 — Supplementary Information. [file 41598_2024_60819_MOESM1_ESM.zip › Supplementary Figures.pdf]

Supplementary Figures

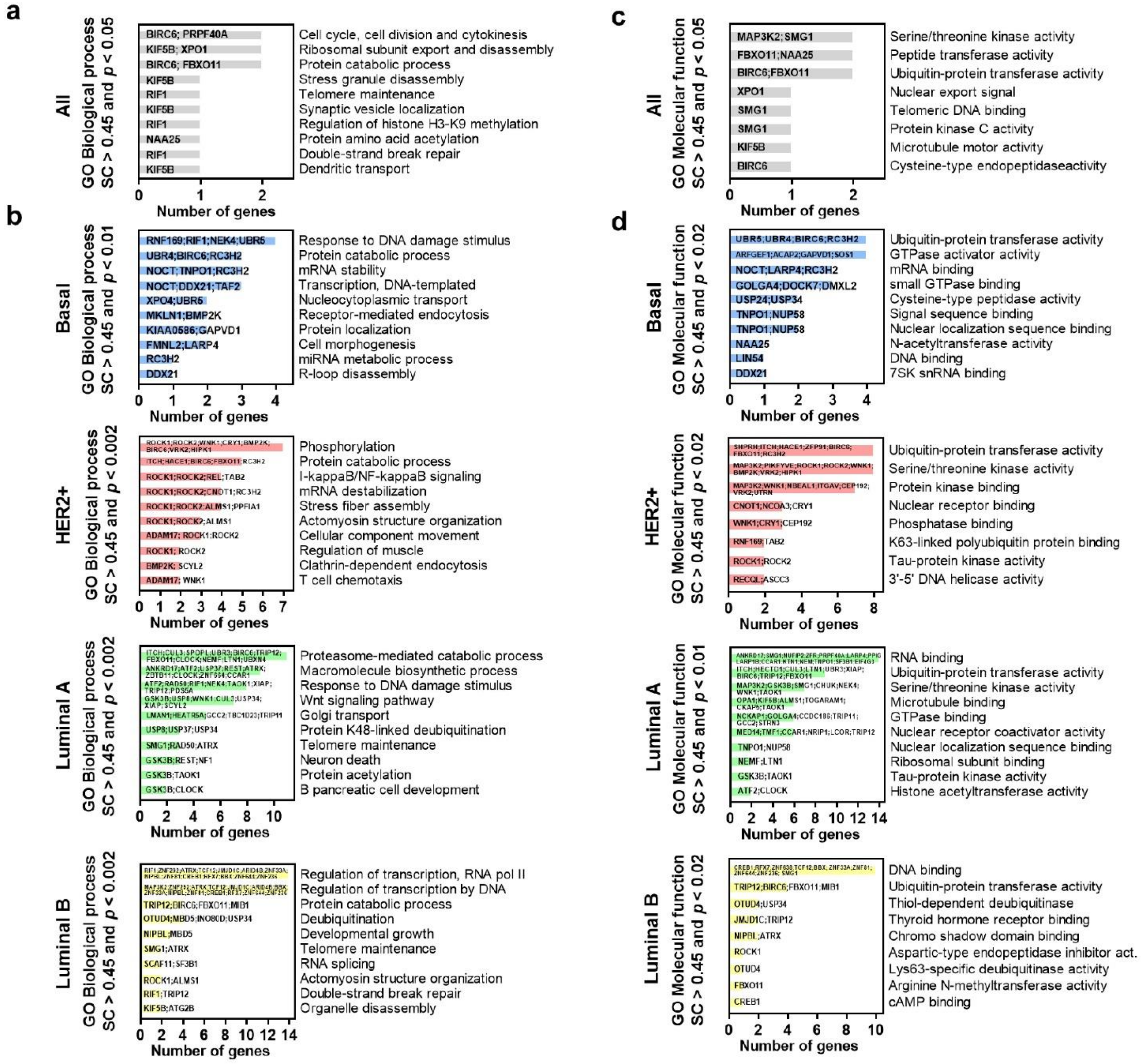

Supplementary Figure 1. Gene ontologies identified by Enrichr of up-regulated genes associated with Tregs infiltration. Biological process ontologies of up-regulated genes included in all breast cancer sub-types (A) and by subtypes (B). Molecular function ontologies of up-regulated genes included in all breast cancer subtypes (C) and by subtypes (D).

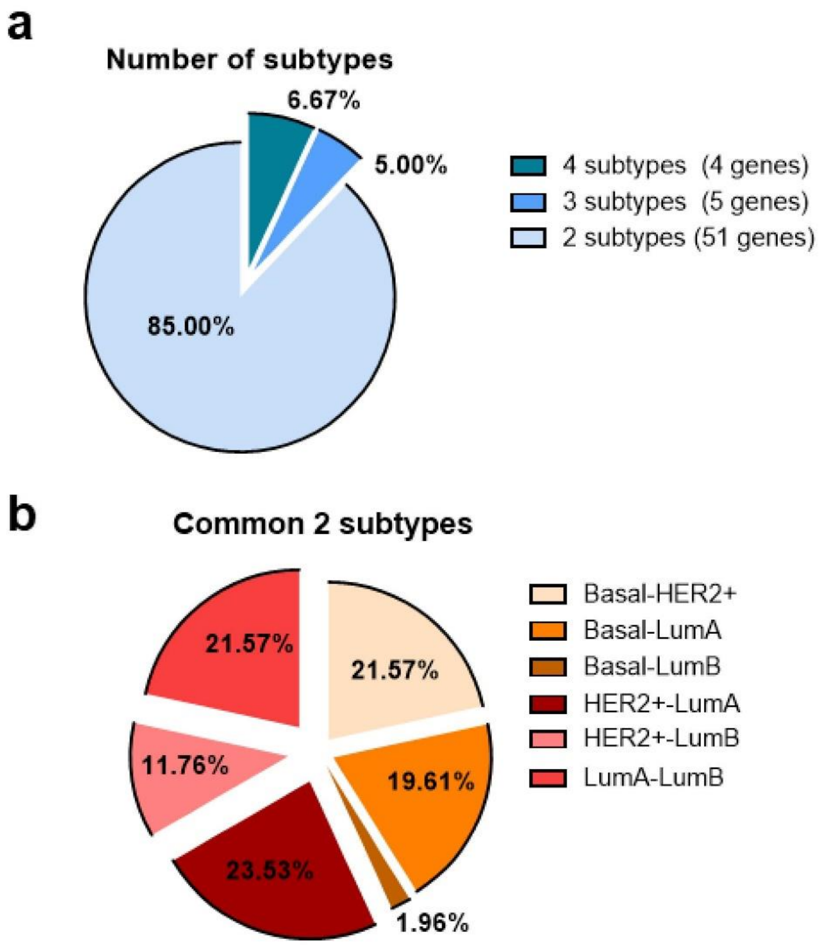

Supplementary Figure 2. Evaluation of surfaceome genes. A. Pie chart with proportion of surfaceome genes by common subtypes. B. Pie chart with proportion of surfaceome genes by common 2 subtypes.

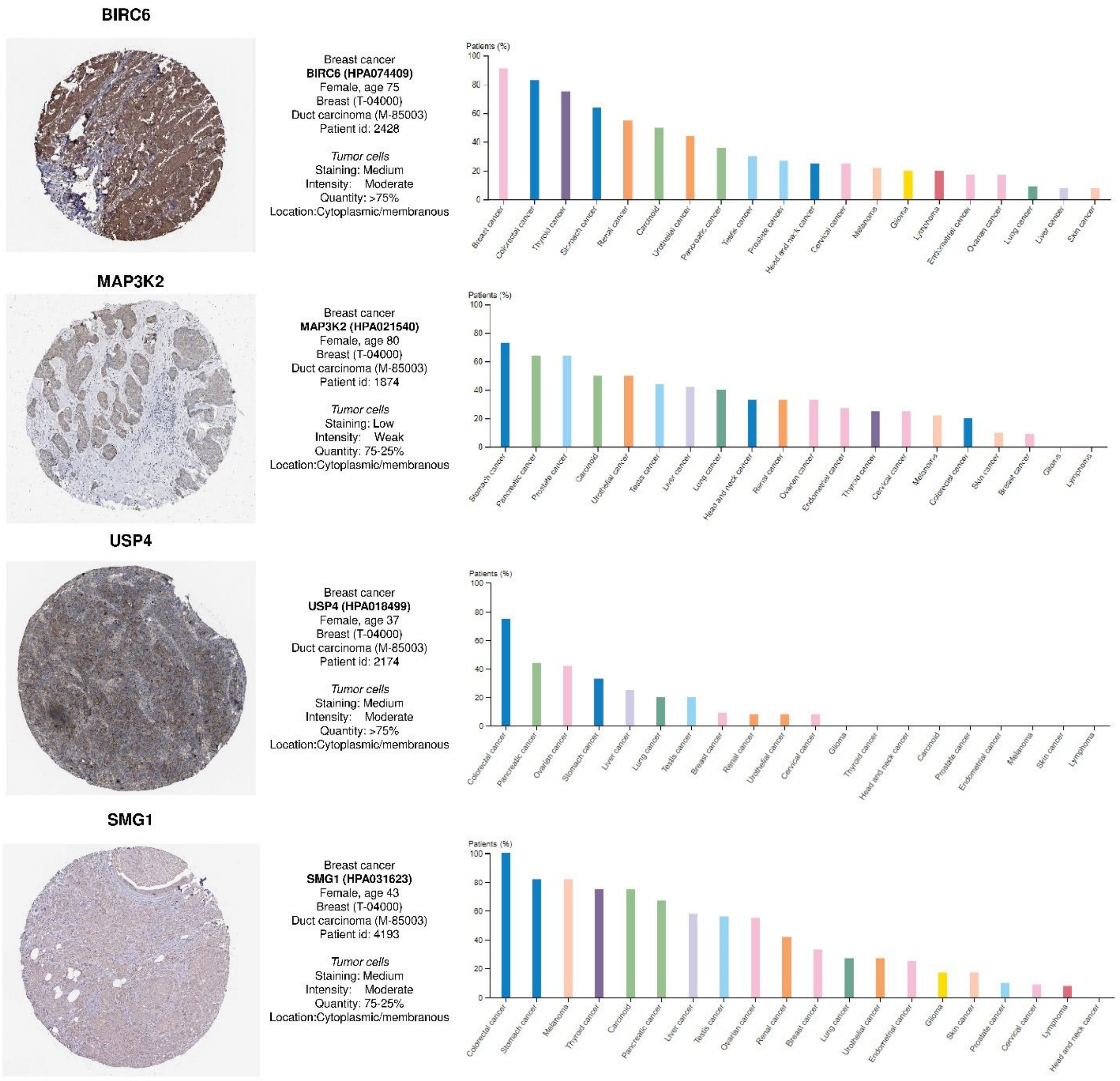

Supplementary Figure 3. BIRC6, MAP3K2, USP4 and SMG1 protein expression summary with an example of a breast cancer immunohistochemistry. This data is included in Pathology section, based on mRNA and protein expression data from 17 different forms of human cancer, together with millions of in-house generated immunohistochemically stained tissue sections images in Human protein atlas (https://www.proteinatlas.org/).

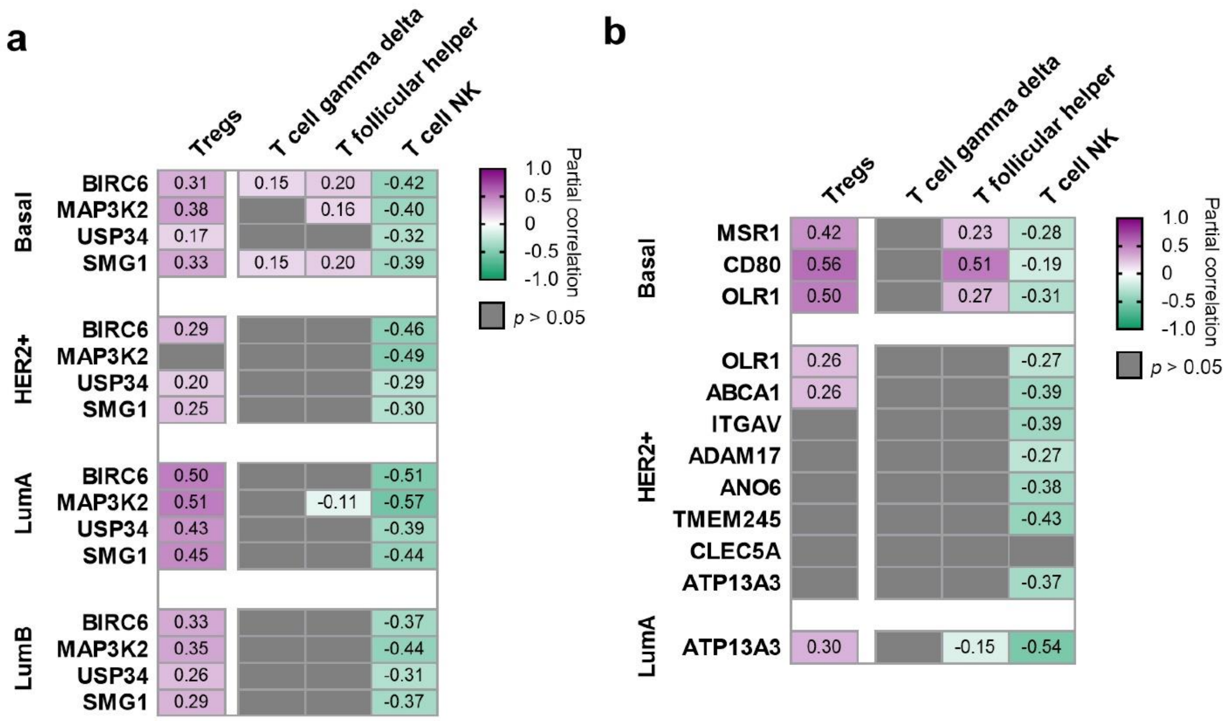

Supplementary Figure 4. Heat map depicting the Pearson correlation coefficient (R) between gene expression, and the presence of different T cells populations by TIMER.

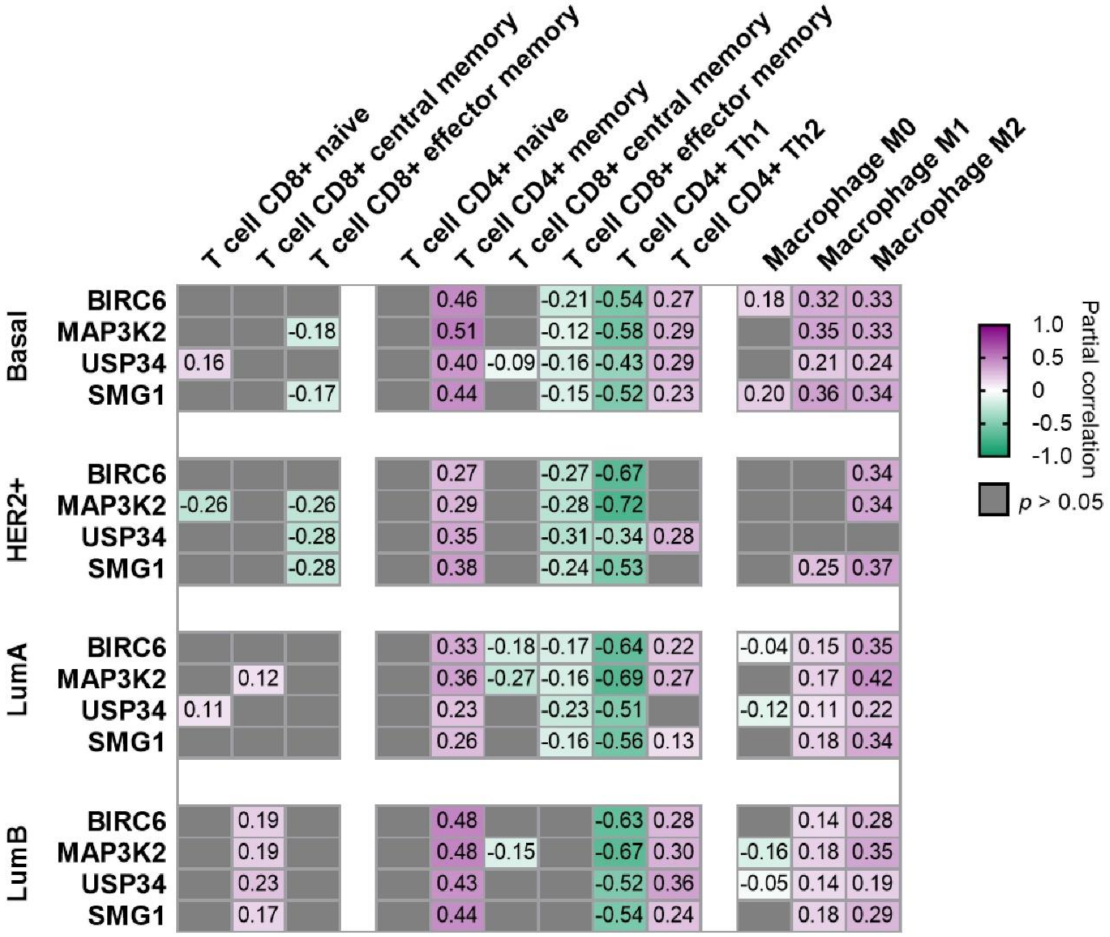

Supplementary Figure 5. Heat map depicting the Pearson correlation coefficient (R) between gene expression, and the presence of different T CD4+, TCD8+, and macrophages populations by TIMER.

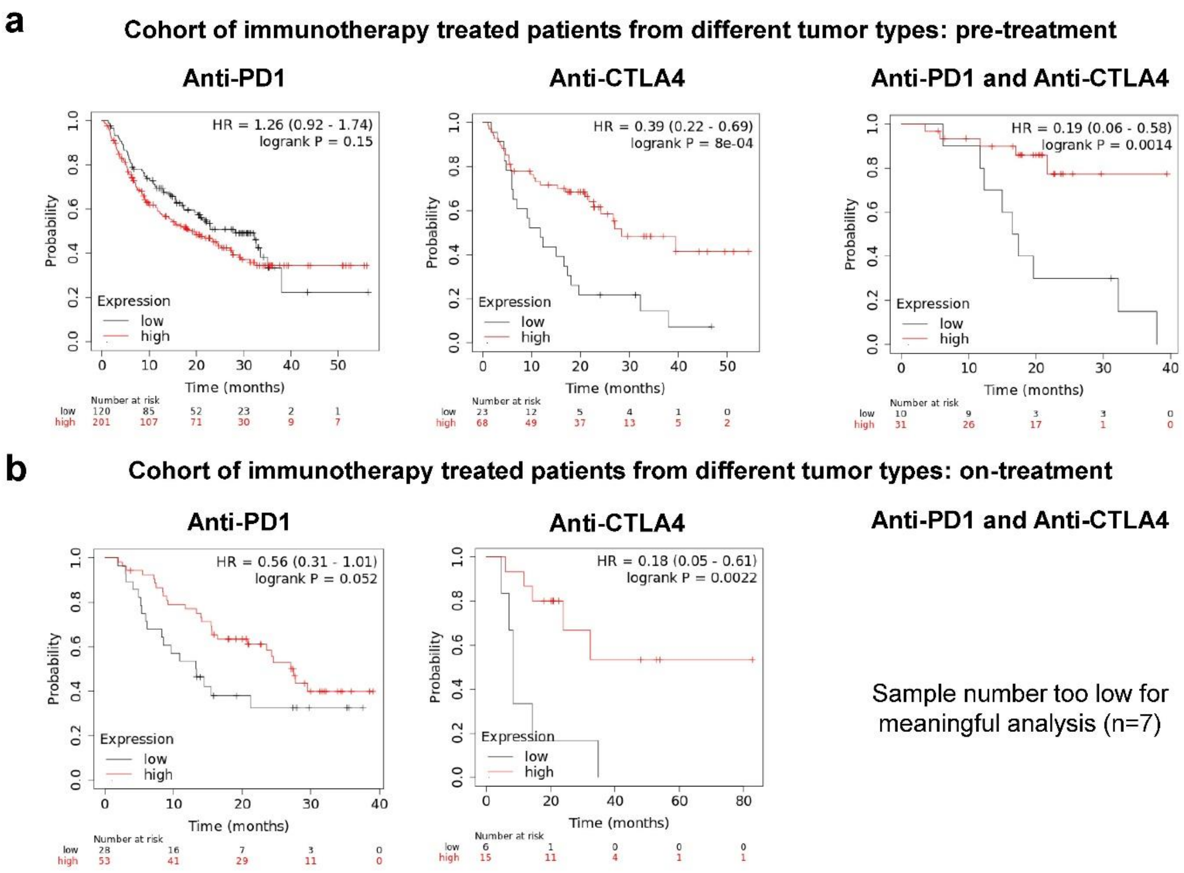

Supplementary Figure 6. Kaplan–Meier survival plots of the association between surface genes mean expression levels and patient prognosis conditionate to anti-PD1, anti-CTLA4, and both treatments. We restricted analyses to pre-treatment samples (A) or on-treatment samples (B).

Anti-CTLA-4 treatment: Ipilimumab only

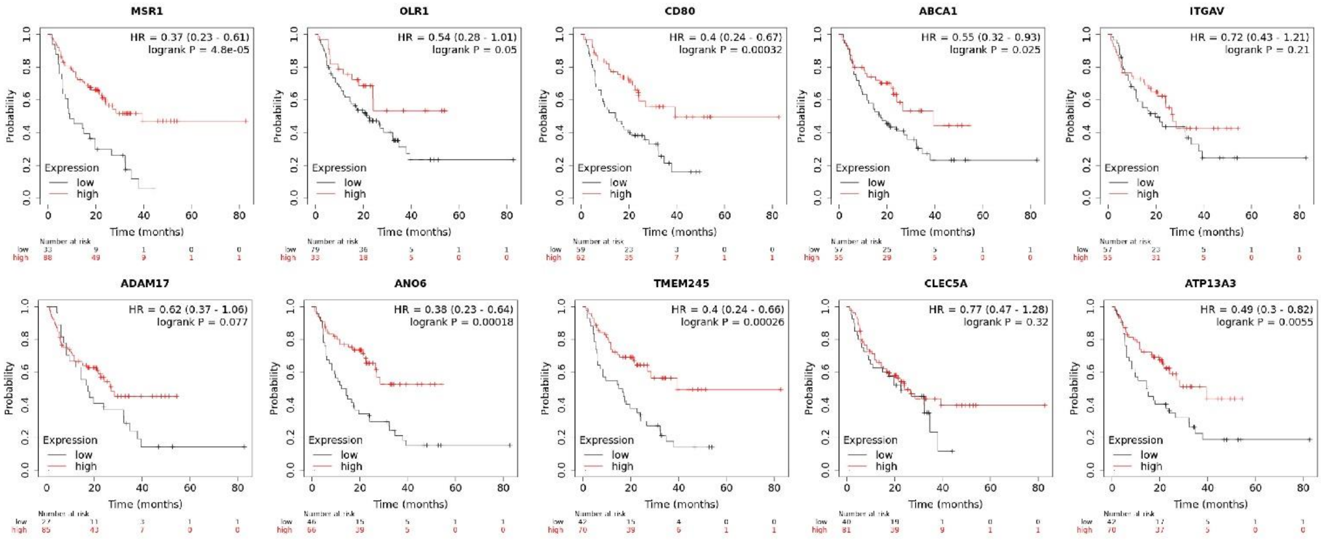

Supplementary Figure 7. Kaplan–Meier survival plots of the association between surface genes individually expressed and patient prognosis conditionate to Ipilimumab anti-CTLA4 treatment for all exploratory cohort.

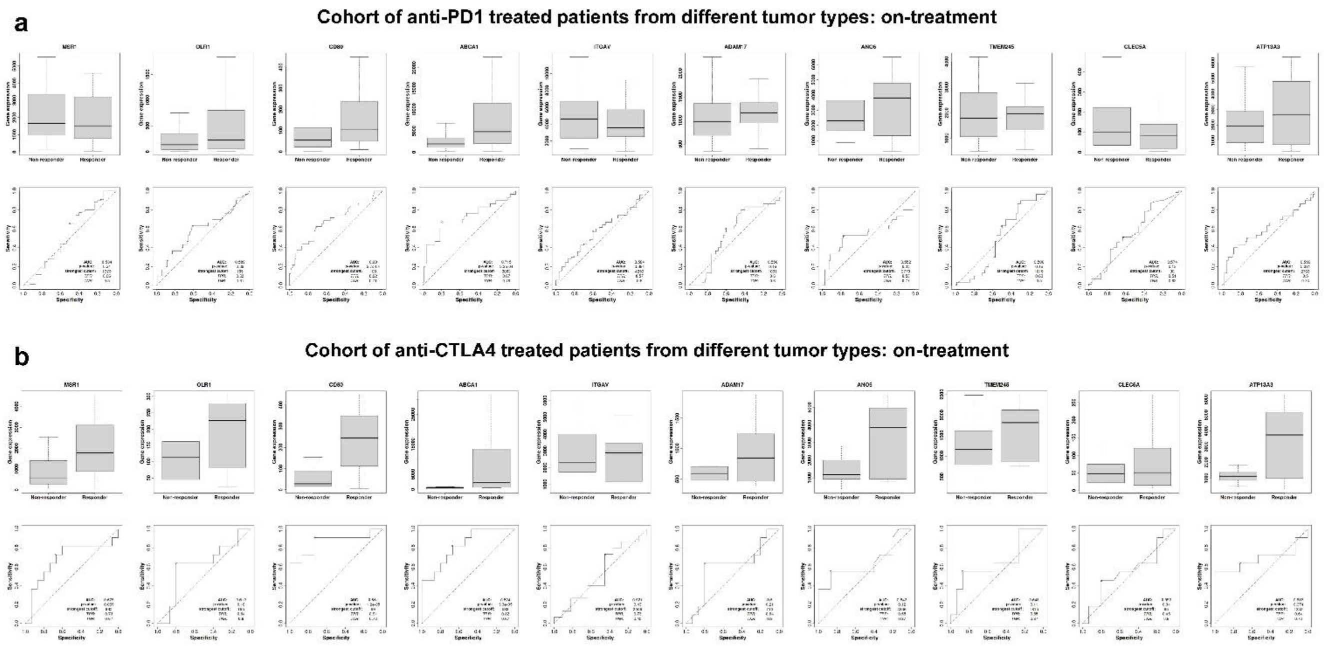

Supplementary figure 8. Surface genes expression related with response to anti-PD1 and anti-CTLA4 treatment. Box-plots of genes validated for Anti-PD1 response in (A) or Anti-CTLA4 response (B) in cancer patients using the pathological complete response database in ROC plotter. Graphs show normalized gene expression in non-responders (NR) and responders (R) patients restricted to on-treatment samples analyses.
