# Supplementary material for: T-reg transcriptomic signatures identify response to check-point inhibitors
Source: Sci Rep. 2024 May 6;14:10396. doi: 10.1038/s41598-024-60819-8 (PMC11074113; doi:10.1038/s41598-024-60819-8)
Supplement: Supplementary file 1 — Supplementary Information. [file 41598_2024_60819_MOESM1_ESM.zip › Supplementary Table legends.docx]

**Supplementary Table legends**

**Supplementary Table 1.** Description of datasets used in this study.

**Supplementary Table 2.** Genes with spearmen correlation > 0.45 by breast cancer subtypes.

**Supplementary Table 3.** Surfaceome genes. Description of biological function of the surfaceome genes obtained by GeneCards.
